# Genome comparison of the epiphytic bacteria *Erwinia billingiae *and *E. tasmaniensis *with the pear pathogen *E. pyrifoliae*

**DOI:** 10.1186/1471-2164-11-393

**Published:** 2010-06-22

**Authors:** Michael Kube, Alexander M Migdoll, Isabel Gehring, Katja Heitmann, Yvonne Mayer, Heiner Kuhl, Florian Knaust, Klaus Geider, Richard Reinhardt

**Affiliations:** 1 Max Planck Institute for Molecular Genetics, htpt group, Ihnestr. 63, D-14195 Berlin, Germany; 2 Julius Kuehn Institute, Institute for Plant Protection in Fruit Crops and Viticulture, Schwabenheimer Str. 101, D-69221 Dossenheim, Germany; 3 Heidelberg Institute for Plant Science, Neuenheimer Feld 360, D-69120 Heidelberg, Germany

## Abstract

**Background:**

The genus *Erwinia *includes plant-associated pathogenic and non-pathogenic *Enterobacteria*. Important pathogens such as *Erwinia amylovora*, the causative agent of fire blight and *E. pyrifoliae *causing bacterial shoot blight of pear in Asia belong to this genus. The species *E. tasmaniensis *and *E. billingiae *are epiphytic bacteria and may represent antagonists for biocontrol of fire blight. The presence of genes that are putatively involved in virulence in *E. amylovora *and *E. pyrifoliae *is of special interest for these species in consequence.

**Results:**

Here we provide the complete genome sequences of the pathogenic *E. pyrifoliae *strain Ep1/96 with a size of 4.1 Mb and of the non-pathogenic species *E. billingiae *strain Eb661 with a size of 5.4 Mb, *de novo *determined by conventional Sanger sequencing and next generation sequencing techniques. Genome comparison reveals large inversions resulting from homologous recombination events. Furthermore, comparison of deduced proteins highlights a relation of *E. billingiae *strain Eb661 to *E. tasmaniensis *strain Et1/99 and a distance to *E. pyrifoliae *for the overall gene content as well as for the presence of encoded proteins representing virulence factors for the pathogenic species. Pathogenicity of *E. pyrifoliae *is supposed to have evolved by accumulation of potential virulence factors. *E. pyrifoliae *carries factors for type III secretion and cell invasion. Other genes described as virulence factors for *E. amylovora *are involved in the production of exopolysaccharides, the utilization of plant metabolites such as sorbitol and sucrose. Some virulence-associated genes of the pathogenic species are present in *E. tasmaniensis *but mostly absent in *E. billingiae*.

**Conclusion:**

The data of the genome analyses correspond to the pathogenic lifestyle of *E. pyrifoliae *and underlines the epiphytic localization of *E. tasmaniensis *and *E. billingiae *as a saprophyte.

## Background

The genus *Erwinia *comprises essentially plant-associated bacteria. Two species, *Erwinia amylovora *and *Erwinia pyrifoliae*, are connected with "pome fruit" diseases, fire blight of apple, pear and some ornamentals and Asian pear blight, respectively [1-3]. Other species were isolated from plant surfaces such as *Erwinia billingiae *and *Erwinia tasmaniensis*. Due to their epiphytic occurrence, they can compete with growth and distribution of *E. amylovora *on flowers and may be applied as antagonists for control of fire blight [4]. Such bacteria have the potential to reduce the use of antibiotics in agriculture for control of the disease. Accumulation of streptomycin resistant strains in pome fruit growing regions in the United States emphasizes the need of new strategies to reduce economical losses of more than $100 million per year for the United States resulting from fire blight [5-10].

In the course of description of pear pathogenic *E. pyrifoliae *strains, isolated in Korea, it became evident that they are related to *E. amylovora*, but distinct for several taxonomic criteria [11]. A pathogen associated with bacterial shoot blight of pear (BSBP) in Japan was also classified as *E. pyrifoliae *[12]. Strain Ep1/96 from Korea is assumed to be a representative for *E. pyrifoliae *and the features are also valid for the strains from Japan.

In dendrograms from 16 S rRNA sequences and in alignments of parts from the house keeping genes *gpd *and *recA*, *E. pyrifoliae *is related to *E. amylovora*, less to *E. tasmaniensis *and in more distance to *E. billingiae *[12].

Several properties of these species can explain their interactions with plants. Pathogens as well as epiphytic bacteria are dependent on the availability of carbohydrates to metabolize them as an energy source. Plants synthesize and transport high levels of sucrose as a main product of photosynthesis. *E. amylovora*, the fire blight pathogen, is specialized on rosaceous plants, which typically also produce sorbitol. Mutants of *E. amylovora *in the sucrose and in the sorbitol metabolism are non-pathogenic [13,14]. Surprisingly, *E. tasmaniensis *lacks the *srl*-operon and is unable to metabolize sorbitol, although the epiphytes were isolated from the apple and pear flora [15,16]. *E. billingiae *was isolated from a similar environment, initially designated as "white *Erwinia herbicola*" and later classified as a novel species [17,18]. These bacteria are also detected in necrotic plant tissue from trees with fire blight (Geider et al., unpublished), but do not share important properties with *E. amylovora *[4]. These include the inability of *E. billingiae *to cause a hypersensitive response (HR) on tobacco leaves, a lack of levan synthesis with a general deficiency to metabolize sucrose [4]. A deficiency in levan formation was also observed for *E. pyrifoliae *[1]. On the other hand, destruction of the host plant tissue, indicated by HR on non-host plants, is an important pathogenicity factor of *E. amylovora *and was also described for *E. pyrifoliae *[1,19,20].

Another important pathogenicity factor of *E. amylovora *and *E. pyrifoliae *is the formation of capsular exopolysaccharide (EPS). Gene clusters encoding functions for amylovoran synthesis of *E. amylovora *and pyrifolan synthesis of *E. pyrifoliae *have been described and mutants created in these genes by transposon and by site directed mutagenesis, which lost virulence [21,22]. Other virulence factors of *E. amylovora *were described to include iron uptake, or defects in amino acid or nucleotide metabolism [23].

Therefore it was of special interest to show, if factors necessary for invasion can be identified in the genome of *E. pyrifoliae *and if their absence in the genome of *E. billingiae *and *E. tasmaniensis *can describe their epiphytic occurrence in plant surfaces.

We determined the complete genome of the pear-pathogen *E. pyrifoliae *strain Ep1/96, which is related to *E. amylovora *and of the non-pathogenic *E. billingiae *strain Eb661 [11,24]. A mixed strategy of pyrosequencing and traditional Sanger sequencing was used to determine both new genome sequences. These data together with the previously published genome sequence of *E. tasmaniensis *provide databases for comparative analysis of virulence factors.

## Results and Discussion

### Genome composition and architecture of *Erwinia billingiae *strain Eb661, *E. tasmaniensis *strain Et1/99 and *E. pyrifoliae *strain Ep1/96

**Figure 1:**
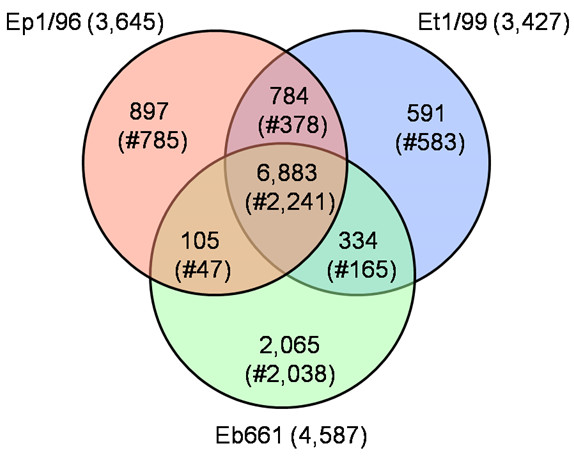
**Venn diagram for the deduced proteins of *E. pyrifoliae *strain Ep1/96, *E. tasmaniensis *strain Et1/99 and *E. billingiae *strain Eb661**. Values were calculated by BLASTCLUST using an identity of >60%, an alignment length of >70% and an e-value of 1e-6 as cut-off. The number of proteins per chromosome is given. The number of clusters (#) is given in brackets representing the non-redundant protein-coding genes per intersection. The overlapping sections indicate shared numbers of proteins. The total number of all deduced proteins of these three species is 11,659.

The genomes of *E. billingiae *strain Eb661, *E. tasmaniensis *strain Et1/99 and *E. pyrifoliae *strain Ep1/96 contain one circular chromosome with a size of 5.1 Mb for strain Eb661, and 4.0 Mb for strain Ep1/96 down to 3.9 Mb for strain Et1/99 [15]. The number of corresponding predicted proteins ranges from 4,587 to 3,427 (Table 1). The distant position of *E. billingiae *strain Eb661 is indicated by the general genome data and corresponds to the phylogenetic position on a different branch in contrast to the other three genomes [12,24]. This classification into two groups is also supported by the estimated numbers of shared genes for the chromosomes of the strains Ep1/96, Et1/99 and Eb661 (6,883 out of 11,659 proteins in total) (Figure 1). The highest amount of unique proteins is present for strain Eb661 with 2,037 proteins (2,065 including paralogs etc.) corresponding to the enlarged chromosome size. This portion encompassing 4% of the deduced protein set encodes a wide additional metabolic repertoire and associated transporters. In contrast, the portion of estimated unique genes for the pathogenic strain Ep1/96 is 785 (897 including paralogs etc.) corresponding to approx. 25% of the proteins encoded on the chromosome.

**Table 1:** Chromosome features for the genomes of *E. pyrifoliae *strain Ep1/96, *E. tasmaniensis *strain Et1/99 and *E. billingiae *strain Eb661.

Strain	Ep1/96	Et1/99	Eb661
Size (bp)	4,026,322	3,883,467	5,100,168

Seq. coverage	34*^1^	11	19*^2^

G+C content (%)	53.4	53.7	55.2

Protein coding (%)	84.9	85.0	87.7

Coding sequences	3,645	3,427	4,587

Average size (bp)	938	964	975

G+C content (%)	54.6	54.9	56.3

Assigned function	2,846	2,676	3,768

Conserved uncharacterized	549	389	515

Uncharacterized	250	362	304

Transposases	90	25	10

assigned as pseudo	57	57	13

rRNA operons	7	7	7

tRNAs	75	81	77

extrachromosomal elements	4	5	2

Sequencing coverage was reached by a 9fold Sanger sequencing and a 25fold coverage with GS20 data for strain Ep1/96 and by a 11fold Sanger sequencing and a 8fold coverage with GS FLX data for strain Eb661.

rRNA operons show 16S-23S-5S order, except one copy within the chromosome of the strains Ep1/96, Ea273 and Et1/99 with the unusual 16S-23S-5S-5S order organization. The 16S-23S-5S-5S operon is missing in strain Eb661, which belongs to a different phylogenetic branch.

**Figure 2:**
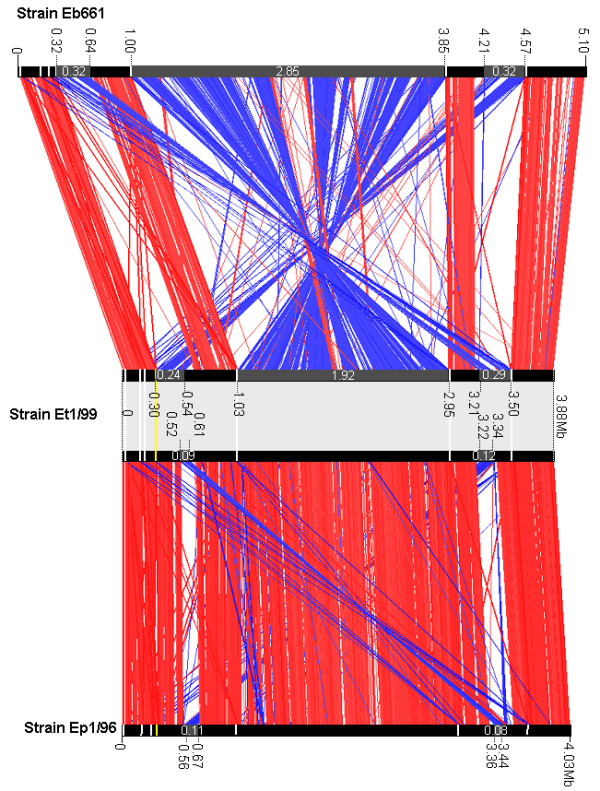
**Chromosome organization of strain Eb661 vs. Et1/99 vs. Ep1/96**. Chromosomes were compared using ACT. Red lines connect homologous regions present in the same orientation while the blue lines connect regions of inverted orientation. Black bars symbolize chromosomes. Localization of the rRNA operons is indicated by white (16S-23S-5 S rRNA organization) and yellow bars (16S-23S-5S-5 S rRNA organization). Translocated and inverted regions are highlighted in grey and the positions on the chromosomes are provided. The estimated size of these regions, which were modulated by several re-arrangements, is noted in megabases.

The number of 7 rRNA operons is constant within the chromosomes, but the presence of one unusual rRNA operon with a 16S-23S-5S-5 S organization is limited to the genomes of strains Ep1/96 and Et1/99. This could provide a genetic marker for these species within the genus *Erwinia*. The occurrence of the unusual 16S-23S-5S-5 S organization within the chromosome is interpreted as the result of a chromosomal recombination event. Large inversions and translocations have occurred frequently during evolution in the genus *Erwinia *(Figure 2). The present conserved synteny between the three genomes is disrupted by large inversions, which indicates two different types of organization. One type is shared by strain Ep1/96 and Et1/99 as well as a second one which is shared by strain Eb661 and strain Ea273 (data not shown). These re-arrangements are thought to be driven by homologous recombination, which often occurs at the rRNA operons [25]. The importance of rRNA operons for these events could be confirmed by the comparison of the chromosomes of strain Eb661 and Et1/99 for the centered inversion, which is suggested to be responsible for the duplication of the 5S-rRNA gene. The comparison of the chromosomes of strain Et1/99 and Ep1/96 indicates the stability of this re-arrangement. Several other translocations and inversions are present especially within two regions not neighbouring to rRNA operons.

However, Figure 2 also illustrates that the larger chromosome size of *E. billingiae *strain Eb661 is mainly based on the presence of single genes scattered over the whole genome and not on the transfer of large gene clusters. The minor influence of phage integrations was estimated by the prediction of integrated regions (258 kb for strain Eb661, 177 kb for strain Et1/99 and 261 kb for strain Ep1/96) and of gene duplication corresponding to the number of potential paralogs (76 for strain Eb661, 57 for strain Et1/99 and 223 for strain Ep1/96). Cumulative GC skew analyses support the weak modulations of the chromosomes by these events by its regular run (Additional file 1).

**Figure 3:**
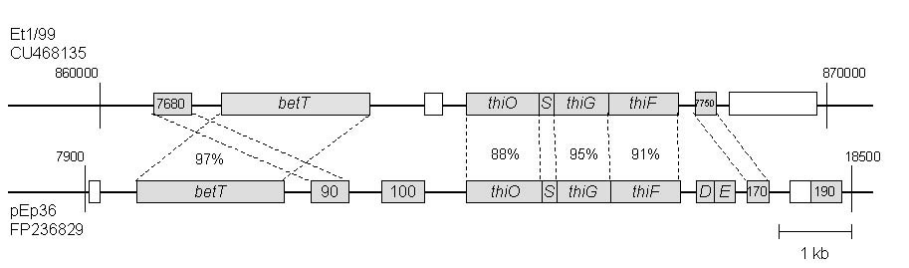
**Alignment of genes involved in thiamine biosynthesis (*thiOSGF*), a toxin-antitoxin system (*stbDE*) and a choline transporter (*betT*) for *E. tasmaniensis*, and *E. pyrifoliae***. The maps show corresponding parts from the chromosome of *E. tasmaniensis *strain Et1/99 and from plasmid pEP36 of *E. pyrifoliae *strain Ep1/96. BlastP results (percentage identity) are shown for related genes (grey boxes). Labels indicating the locus tag number are given within the grey boxes, if no gene name is assigned. White boxes indicate genes without similarity. The proteins for thiamine biosynthesis (*thi*) and the flanking regions are highly related and the genes are in conserved order. The *stbD *and *stbE *genes of pEp36 encode for a toxin-antitoxin system. A comparison for genes in this region was done before for the *E. amylovora *plasmid pEA29, the *E. pyrifoliae *plasmid pEP36 and plasmid pEJ30 from the Japanese *E. pyrifoliae *strain Ejp557 [89]. Locus tags of the genes are abbreviated for this overview.

The exchange of genetic material mainly depends on the presence of extrachromosomal elements. The number of plasmids ranges from two for Eb661 to five in strain Et1/99 (Table 2). A deviating GC content compared to the chromosome suggests an unrelated origin for pET35 and pET49. Several plasmids in the genus *Erwinia *show the potential for a conjugal transfer such as pEB170 of *E. billingiae *strain Eb661, the plasmids pET35 (CU468130), pET45 (CU468132), pET46 (CU468133), pET49 (CU468131) of *E. tasmaniensis *strain Et1/99 and pEL60 of *E. amylovora *strain Leb66 (AY422214), an untypical isolate from Lebanon. Plasmids pEp05 and pEt46 carry *mob *genes and may contain an oriT to be mobilized by Tra proteins of other plasmids. The species *E. amylovora*, *E. pyrifoliae*, and *E. tasmaniensis *share *thiO *(glycine oxidase), *thiS *(sulfur carrier protein), *thiG *(thiazole synthase) and *thiF *(adenylyl transferase) in conserved order. These genes are located on plasmids of *E. pyrifoliae *and *E. amylovora *and on the chromosome of *E. tasmaniensis*. A potential gene flow is indicated by the high identities between the sequences on the nucleotide level and the amino acid level of at least 87% (Figure 3). *E. amylovora *genes for thiamine metabolism are located on plasmid pEA29. Plasmidfree strains of *E. amylovora *show reduced virulence and reduced growth in minimal medium without thiamine [26,27]. The choline transporter protein BetT is encoded in all three species and may help to protect bacteria against osmotic stress [28]. We detected homologies for *stbD *and *stbE *in the sequences of pEp36 but not for *E. tasmaniensis *strain Et1/99. StbD and StbE are plasmid stability proteins and code for a toxin-antitoxin system, which is widespread throughout pathogenic bacteria [29].

**Table 2:** General features of the determined extrachromosomal elements of *E. pyrifoliae *strain Ep1/96, *E. tasmaniensis *strain Et1/99 [15] and *E. billingiae *strain Eb661.

Strain	Ep1/96				Et1/99					Eb661	
Plasmid tag	**pEp2.6**	**pEp03**	**pEp05**	**pEp36**	**pEt09**	**pEt35**	**pEt45**	**pEt46**	**pEt49**	**pEb102**	**pEb170**

Size (bp)	2,590	3,070	4,955	35,909	9,299	35,494	44,694	46,159	48,751	102,323	169,778

G+C (%)	44.9	49.0	53.2	49.9	47.1	40.0	50.8	49.0	43.7	51.7	52.3

Protein coding (%)	66.6	58.9	77.3	71.9	87.9	86.5	87.0	63.5	83.9	85.4	78.7

Coding sequences	6	4	5	37	7	42	46	39	61	114	220

Average size (bp)	287	452	766	698	1,168	731	845	752	670	766	607

G+C content (%)	43.9	47.0	52.2	52.7	47.2	40.1	51.8	49.5	43.6	53.0	54.1

Assigned function	1	2	4	26	6	23	29	25	35	54	111

Conserved uncharacterized	1	2	1	7	1	3	1	5	5	40	15

Uncharacterized	4	-	-	4	-	16	16	9	21	20	94

Transposase(s)	-	-	-	7	-	-	-	2	-	1	6

mobilization or transfer gene	-	-	*mob*	-	-	*tra*	*tra*	*mob* /*tra*	*tra*	-	*tra*

Pathogenicity of several *Erwinia *species is probably based on a large set of modules (Table 3).

**Table 3:** Genetic elements with potential impact to virulence of pathogenic erwinias were summarized and the presence of the key genes is indicated for the analysed genomes (for details please see Additional file 3).

Functional context	Description	Key genes	Ep1/96	Et1/99	Eb661
**Secretion systems and effectors**					

hrp/hsv/dsp cluster (T3SS)	Hrp-associated enzymes (HAE)	*hrpK, hsvABC*	+	-	-
	
	Hrp/hrc secretion/translocation pathway	*hrcUTSRQ, hrpPO, hrcN;hrpQIJLXYSAB; hrcJ, hrpDEFG, hrcC, hrpTV*	+	+	-
	
	Hrp elicitor/effector region (HEE)	*hrpN, orfABC, hrpW, dspEF*	+	+	-

Salmonella SPI-1-like T3SS	Invasion proteins	*invHFGEABCIJ*	(+)	(+)	-
	
	Surface presentation of antigens (SPA)	*spaOPQRST*	(+)	(+)	-
	
	Cell invasion proteins	*sicA, sipBCDA, sptP, prgHIJK, orgA*	(+)	(+)	-
	
	Invasion plasmid antigen (IPA)	*ipaBCDA*	-	-	-

T6SS	T6S proteins and effectors	various	+*	+*	+*

Effector and virulence associated	putative operon involved in virulence	*srfABC*	+	+	+
	
proteins	Outer membrane protease	*sopA*	+	-	-
	
	Maintenance of virulence plasmid	*mvpT*	+	+	-
	
	Virulence protein; regulator for acidic stress; integral membrane protein, outer membrane protein	*msgA, phoPQ, mviN, ompX*	+	+	+
	
	Outer membrane invasion protein	*pagC*	+	-	+
	
	Virulence associated protein	*virK*	-	+	+

**Metabolism**					

Capsular polysaccharide systems	Capsular polysaccharide- biosynthesis	*amsGHIABCDEFKL, galFE/cpsGHIABCDEFKL, galFE*	+	+*	+*
	
	Putative cps biosynthesis system	*ymcABC*	+	+	+
	
	Regulators	*rcsA, rcsB, rcsC, rcsF*	+	+	+

Levan-metabolism	Levan synthesis	*lsc*	-	+	-
	
	Regulators	*rlsA, rlsB, rlsC*	+	+	-

Sorbitol-Operon	Sorbitol uptake, metabolization	*srlAEBDMR*	+	-	+

Sucrose-Operon	Sucrose uptake, metabolization	*scrKYABR*	+	+	-

Non-ribosomal peptide synth.	Suggested phytotoxin	*eppT*	+	-	-

**Adhesion & extracellular factors**					

Cell-influencing factors	Necrosis factors	*cnf1, cnf2*	+	+	-
	
	Proteases	*ptrA, ptrB*	+	+	+
	
	Siderophores	*foxR, dfoA, alcA*	+	+	(+)

The presence is weighted by three criteria: "+" completely present, "(+)" present in parts and "-" absent. An extended gene content by the presence of additional genes is marked by "*".

We focussed the analyses on the genetic environment encoded within the genomes with respect to secretion, production of exopolysaccarides, sorbitol and sucrose metabolism as well as on the presence of miscellaneous genes with potential impact to the virulence for the pathogenic erwinias.

### Secretion systems

The ability to secrete effector proteins and to colonize host plant tissue represents very important features. Differences in chromosome content were also identified by mapping the deduced proteins from the non-pathogenic strains on the chromosome of *E. pyrifoliae *strain Ep1/96 as well as by one by one comparison (Additional file 2, **Figure S2A-E**; Additional file 3). The data for several combinations are arranged in Additional file 2 and several secretion systems are classified in Additional file 3 for their presence or absence in the investigated genomes.

Common secretion systems of the *Enterobacteriaceae *such as the *sec*-independent (Type 1) and *sec*-dependent (Type 2) secretion systems have been identified in *Erwinia *species that fulfil vital functions, e.g. export of extracellular proteins for nutrient acquisition [30]. Among them, one of the best studied are the genetically and structurally conserved type III secretion systems (T3SSs), which were found to be crucial for delivery of proteins acting as pathogenicity factors into the extracellular space or the cytoplasm [31,32]. Target cells and secreted proteins are of broad range and host-specific.

The primary T3SS in *Erwinia *species is composed of the *hrp/hrc*-gene-cluster and two flanking regions (Hrp elicitors and effectors [HEE] and Hrp-associated enzymes [HAE]), which contain effector proteins and enzymes involved in systemic virulence [15,33]. Like in *E. amylovora *this assembly could also be identified in the pathogenic *E. pyrifoliae *strain Ep1/96, and clearly marks a difference to the system of the non-pathogenic *E. tasmaniensis *strain Et1/99 without the HAE region and *E. billingiae *strain Eb661, which contains no homologs to a T3SS [15]. Nevertheless, the *hrp/hrc*-clusters of *E. pyrifoliae *and *E. tasmaniensis *show almost conserved synteny. Slight differences are *orfU1 *(Acc. No. AAD24685) and *orfU2 *(Acc. No. AAD24686) of *E. amylovora *with similarity to genes coding for hypothetical proteins in *Helicobacter pylori *[33]. The products of those CDS may represent specific components of the *E. amylovora *T3SS.

A second gene cluster similar to the HAE region could be identified in *E. pyrifoliae*. The two genes *hsvA *and *hsvC *show a high similarity on amino acid level of 86% and 66%, respectively, to their counterparts in the *hrp/hrc*-T3SS. However, a corresponding *hsvB*-gene was not found in this cluster but a sequence coding for a putative capsular exopolysaccharide synthesis protein.

An incomplete T3SS similar to the *Salmonella *pathogenicity island 1 (SPI-1) and also found in the insect endosymbiont *Sodalis glossinidus*, may have a distinct function in *Erwinia *species [15,34]. This region, spanning about 20 kbp in *E. pyrifoliae *and *E. tasmaniensis*, contains most of the invasion- (*invFGEABC*), surface presentation of antigens (*spaOPQRS*) and invasins with the associated chaperone-genes (*sicA, sipB, sipD*) as well as those encoding the needle complex (*prgHIJK, orgA*). However, three CDS for conserved hypothetical proteins were found replacing *sipC*, an essential invasion gene and *invIJ*, encoding putative effectors [35,36]. Furthermore, the genes *sipA*, *iacP, sicB, sptP, iagB, hilA, orgBC *and *hilC*, which constitute mainly regulatory components, are lacking. The gene content and order is highly conserved between *E. pyrifoliae *and *E. tasmaniensis*. A similar island was not found in *E. billingiae*. However, it remains unclear, if this T3SS is operative because of the incompleteness in comparison to the SPI-1 and the replaced genes. Recent results from pathogenicity tests on immature pears with SPI-1-like mutants of *E. amylovora *indicate that it is not essential for pathogenicity [37]. Even for *Salmonella typhimurium *it was shown, that only the initial infection stages are affected in mutants while they remain pathogenic when applied by different routes [38].

Only few CDS for putative effector proteins could be identified in the erwinias. Most of those proteins are thought to affect or to be secreted by the T3SS. The suggested effector SrfC of *Pectobacterium carotovorum *subsp. *atrosepticum *is also thought to be exported by T3SS [39]. *E. billingiae *carries the *srfABC *gene cluster like the other three erwinias, but is lacking the instrumentation for a T3SS. The function of SrfC remains unclear, in consequence.

Both pathogenic erwinias possess coding sequences for the SopA protein, which has been characterized as an effector-like protein in *Salmonella *influencing the inflammatory response of mammalian hosts [40]. This protein is translocated via the *Salmonella *T3SS on the SPI-1 into eukaryotic cells and seems to be necessary for full virulence [41]. Since a similar T3SS has been identified in the pathogenic erwinias, one could assume that the SopA effector has a particular role in pathogenicity of those bacteria in plants. It could influence proteins in the plant cell to alter defence response to bacterial invasion. Another putatively SPI-1 dependent system found in the four *Erwinia *species is composed of the small operon *srfABC*, which seems to be regulated by SPI-1 activation [42]. Repression is accomplished by RcsB and PhoP, whose coding sequences could be identified in the erwinias. For several effectors the SPI-1 related T3SS may has a special function, which is different to the *hrp/hrc*-T3SS but probably not essential for virulence because it is also present in the non-pathogenic species *E. tasmaniensis*. This would be in accordance to rececently published results on SPI-1 mutants of *E. amylovora *[37].

The gene *virK*, which is secreted by the second *Salmonella *T3SS found on the pathogenicity island 2 (SPI-2) and regulated by the *phoPQ*-genes, is a pathogenicity factor of *Salmonella *sp. [43]. A coding sequence for VirK was identified in *E. tasmaniensis *and *E. billingiae *but not in the pathogenic erwinias. A possible reason could be the missing secretion system for this protein, which led to the loss of the gene in the process of specialization. The T3SS share a wide homology that could support secretion by the other systems found in the *Erwinia *species [30]. A simple protein export machinery is built by the Type V secretion system (T5SS), which is found in various bacteria [44]. The main domains, a leader sequence and an extracellular effector domain, and an outer membrane export channel, are sometimes encoded on one sequence and constitute one protein. Because of the self-assembly and -export they were termed autotransporters. Another strategy, dubbed two-partner secretion, is characterized by separate expression of leader-effector protein and the leader-channel protein. Most effector proteins are involved in adherence, invasion and degradation [44]. The non-pathogenic *E. billingiae *is the only species where we identified genes for corresponding autotransporters (EbC_25980, EbC_37340). They show similarities to the AidA domain family, which is mainly present in enteropathogenic bacteria, and pertactin, an autotransporter found in *Bordetella *sp., respectively. The primary role of the afore mentioned proteins is adherence to target structures. It may be possible that they substitute the function of missing fimbrial parts found in the other *Erwinia *species, a difference to strains Ep1/96 and Et1/99.

An emerging class of secretion systems, possibly related with pathogenicity, is the recently identified Type VI secretion system (T6SS) in Gram-negative bacteria [45]. Analyses revealed one large (EpC_06160-EpC_06400, vgrG) and one small cluster (EpC_19520-EpC_19550) in *E. pyrifoliae*. Both clusters are present in *E. tasmaniensis *and *E. billingiae*, but show variations in gene content (Additional file 4). Most of the still uncharacterized genes are conserved within the clusters. Functions were assigned for the putative regulator Fha (involved in phosphorylation), the membrane associated proteins such as Lip (outer membrane lipoprotein), IcmF and DotU (inner membrane proteins), ClpV (ATPase) and Hcp and VgrG [46,47]. The proteins Hcp and VgrG are secreted, Hcp building a tube-like structure for effector delivery, while VgrG may be an effector-activator or an effector itself [45]. One CDS, found in *E. pyrifoliae *(EpC_06280) and *E. billingiae *(EbC_05860), of the larger cluster codes for a putative exported protein, which shows similarities of 50-65% to a protein of other plant-pathogenic bacteria such as *Pectobacterium atrosepticum *(syn. *Erwinia carotovora *ssp. *atroseptica*) and *Pseudomonas syringae *pv. *tomato*. In case this exported protein has an effector function, it would match the previous results, that *E. tasmaniensis *is missing many other effector proteins [15]. Whether the secretion systems have an influence on pathogenicity is undiscernible so far, since only a rudimentary instrumentation was found in *E. amylovora*. Plant invasion, which was not confirmed for *E. tasmaniensis*, may be an important requirement for the function (i.e. translocation of proteins into dedicated host cells).

For the T6SS essential gene content, function assignment and structural determination is not well advanced. Most information exist for animal pathogens, but also plant pathogens may use T6SS [48]. Nevertheless, the intrinsic role of the T6SS beside T3SS and/or T4SS has yet to be determined.

### Genetics of EPS synthesis by *E. pyrifoliae *and *E. billingiae*

Several metabolic factors are considered to play an important role for causing disease in *Erwinia*-infected plants including synthesis of exopolysaccharides (EPS) i.e. amylovoran or related products and levan production as well as metabolism of sorbitol and sucrose [49].

The capsular EPS of *E. amylovora *is amylovoran, which apparently modulates recognition of the bacteria by plant defense mechanisms and is a main pathogenicity factor [50]. The gene cluster for EPS-synthesis of pyrifolan by *E. pyrifoliae *also consists of 12 CDS (Additional file 3) with two adjacent genes for precursor synthesis. The encoded proteins are at least 85% similar and have a conserved order for *E. pyrifoliae *and *E. amylovora*. The repeating units of amylovoran and pyrifolan have the same sugar composition and identical linkages except a missing second side chain of glucose for pyrifolan [22].

No EPS has been identified so far for *E. tasmaniensis*, but there is a gene cluster for synthesis of capsular polysaccharide on the chromosome [15]. *E. billingiae *possesses similar genes but produces an EPS. In alignments of Cps proteins from *E. pyrifoliae *and *E. billingiae*, CpsF (WceF) have a remarkable divergence, which may indicate specific functions for processing and assembly of the repeating units of capsular EPS for both species.

*E. amylovora *produces another EPS, levan, which serves as quickly generated shield against recognition by plant defence reactions [51]. The secreted levansucrase, encoded by *lsc*, cleaves sucrose into glucose and fructose, which is subsequently polymerized to levan [52]. Levan is not strictly necessary for virulence of *E. amylovora *[51]. Also the non-pathogenic *E. tasmaniensis *possesses an *lsc *gene and produces levan [15]. *E. pyrifoliae *lacks the *lsc *gene, but an *orf *(EpC_17920) coding for a protein similar to levanase was identified [22]. Levanase belongs to the β-D-fructofuranosidases and can also cleave inulin and sucrose [53]. Therefore, the enzyme could provide nutrients by cleavage of fructans in plant tissue, and may degrade levan from synthesizing bacteria, if it is secreted by *E. pyrifoliae*.

### Sorbitol and sucrose metabolism

A dominant carbohydrate in rosaceous plants is the transport sugar alcohol sorbitol. In case of *E. amylovora *the proteins for its metabolization are encoded by the genes *srlAEBDMR*, which could also be identified for *E. pyrifoliae *and *E. billingiae *but not in the genome of *E. tasmaniensis *[13,15]. Virulence assays of *E. amylovora srl*-mutants showed reduced symptoms on apple seedlings but only weak effects on pear slices due to their low content of sorbitol. The content of sorbitol is high in leaves and in transport tissue, and its amount varies during plant development [54].

**Figure 4:**
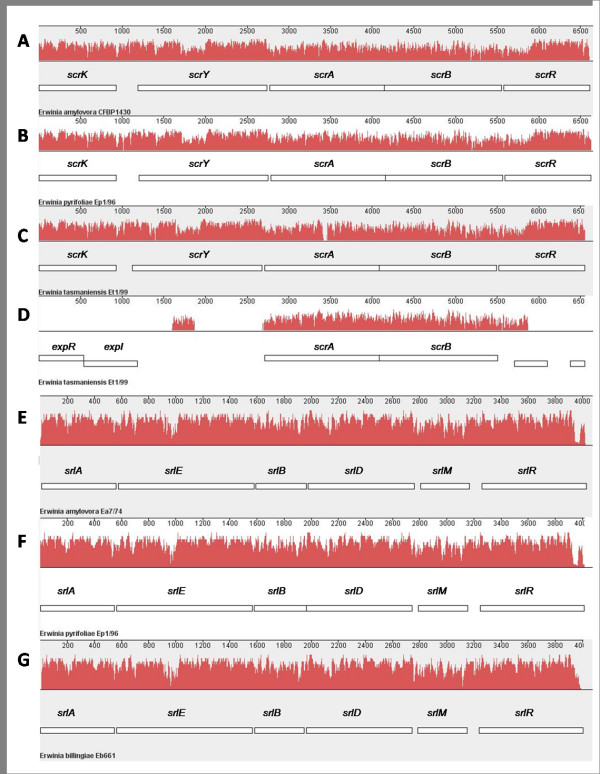
**Alignment of the sucrose and sorbitol operons from *E. amylovora*, *E. pyrifoliae *and *E. tasmaniensis *with Mauve v.2.3.0**. The similarity profile of the alignment in the progressive mode is shown in red [55]. The height of the bars corresponds to the average level of conservation in the sequence. White boxes indicate genes. The alignment of the sucrose operons (A-D) comprise nucleotide sequences of *E. amylovora *strain CFBP 1430 (A; Acc. No. AJ250722, positions 230 6950), *E. pyrifoliae *strain Ep1/96 (B; Acc. No. FP236842, positions 2206738- 2213359) and *E. tasmaniensis *strain Et1/99 (C; Acc. No. CU468135, positions 2119695- 2126241). The genes *scrA *and *scrB *are duplicated in the genome of *E. tasmaniensis *Et1/99 (D; positions 1064052- 1070604). There is a continuous homology in the sucrose operons shown in the similarity profiles. Alignment of the sorbitol operons (E-G) comprises nucleotide sequences of *E. amylovora *strain Ea7/74 (E; Acc. No. Y14603, positions 276- 4479), *E. pyrifoliae *strain Ep1/96 (F; Acc. No. FP236842, positions 668798- 672810) and *E. billingiae *strain Eb661 (G; Acc. No. FP236843, positions 2907872- 2911878).

Sucrose is another important transport sugar in plants. Mutants of *E. amylovora *in the *scr *operon are non-virulent [14]. The disaccharide is actively transported into the cells and subsequently cleaved. Besides the repressor gene *scrR*, four genes encoding steps in sucrose metabolism are present in *E. amylovora*, *E. pyrifoliae *and *E. tasmaniensis*. The genes *scrKYABR *are located in an operon for these three species, but *E. tasmaniensis *carries a second copy of *scrAB *[15]. Alignments with MAUVE of the *srl *regions of *E. pyrifoliae *and *E. billingiae *as well as the *scr *region of *E. pyrifoliae *and *E. tasmaniensis *show a remarkable similarity for these gene clusters including the second *scrAB *copy in the genome of *E. tasmaniensis *(Figure 4) [55]. This region does not completely comprise the *scrAB *gene cluster, but is extended to 400 bp downstream of *scrB *and to a partial similarity at 1000 bp upstream of *scrA*.

An interesting feature of *E. billingiae *is its possible ability to metabolize xylitol. This sugar alcohol is widely distributed in nature and can interfere with bacterial activities [56]. *E. billingiae *may degrade xylitol to avoid interference with its growth and to use it as a carbon source despite its low nutritional value. A similar gene is missing in the genomes of *E. pyrifoliae *strain Ep1/96 and the *E. tasmaniensis *strain Et1/99. On the other hand, *E. tasmaniensis *strain Et2/99 can grow with xylitol as carbon source [16].

### Miscellaneous determinants with potential impact to virulence of pathogenic erwinias

A striking difference between the pathogenic species *E. pyrifoliae *and the non-pathogenic *E. tasmaniensis *is the existence of a nearly complete Type I fimbrial gene cluster in the latter one (Additional file 3). Enterobacterial Type I fimbriae are implied to be involved in cell attachment and adhesion to surfaces leading to biofilm formation [57]. Beside being a virulence determinant the fimbriae may therefore be a tool of epiphytic bacteria for colonization and protection against environmental effects. Without production of a capsular polysaccharide by *E. tasmaniensis*, the fimbriae may replace the capsules to allow bacterial aggregation [15]. This is supported by the lack of a Type I fimbrial gene cluster in *E. billingiae*, which produces EPS and may thus not depend on fimbriae. On the other hand, the existence of Type I fimbriae may induce plant defence responses comparable to the effect of FimH on mammalian cells [58].

While the distribution of Type I fimbriae genes seems to differ, all *Erwinia *species except *E. billingiae *possess a comparable incomplete instrumentation of K88 (F4) fimbriae genes. These fimbriae were found in enterotoxigenic *E. coli *strains and identified as virulence factors, to determine attachment to specific receptors of intestinal cells [59]. The cluster, build up with *faeBCDEFGHIJ*, could be reconstructed except for the regulator FaeB and the component FaeJ, without known expression and function [60]. However, a CDS coding for a possible regulatory protein was found in *E. pyrifoliae *instead of FaeB, leading to the assumption that functional F4 fimbriae participate in adhesion of these species. There are no hints if they are related to pathogenicity.

Another gene cluster first described in enterotoxigenic *E. coli *strains and also found in the erwinias forms the class 5 fimbriae with the surface antigen CS14 in the *csuABCDE*-operon [61]. They are involved for *E. coli *in attachment to specific intestinal epithelial cells. Whether F4 and class 5 fimbriae have a cumulative effect by adhering to receptors of the same cells or aim at different cells and have specific functions is difficult to determine on the molecular level. Since both, pathogenic and non-pathogenic, *Erwinia *species possess these genes, their role in pathogenicity is unclear.

Furthermore, genes for the global regulator of fimbrial expression, the heat-stable nucleoid-structuring protein (*hns*), are present in the erwinias [59]. In *E. amylovora*, *E. pyrifoliae*, *E. billingiae *and *E. tasmaniensis*, copies of an *hns *gene are located on the chromosome and also on a plasmid. *E. tasmaniensis *carries two chromosomal *hns *genes. The global regulator also affects EPS synthesis and the protein binds to the *lsc *promoter region of *E. amylovora *[62].

Strain Eb661 encodes several matrix components necessary for biofilm formation. Genes for cellulose synthesis (*bsc*- and *bcs*-operon) were identified as well as a CDS for the surface protein BapA. This instrumentation is similar to biofilm producing *Salmonella *species [63]. Several potential adhesion factors such as hemaglutinin-like proteins may support surface colonization [64].

The three *Erwinia *species share a common set of genes for proteases and siderophore production, which are important virulence factors in soft rot erwinias [65]. Two proteases (PtrA, PtrB) and siderophores (encoded by *foxR*, *dfoA*, *alcA*) may help in colonization and acquisition of nutrients [15,66]. Interestingly, the protease operon for the secreted enzyme PrtA, connected with virulence of *E. amylovora*, is not represented in the genomes of *E. pyrifoliae*, *E. tasmaniensis *or *E. billingiae *[67].

Furthermore, virulence factors such as the cytotoxic necrotizing factors Cnf1 and Cnf2 were identified. These proteins were connected with pathogenic *E. coli *strains to alter signal transduction resulting in modified cell morphology and cell cycles as well as decreased phagocytosis of the bacteria [68,69]. Cnf1 was shown to inhibit apoptosis, which may help bacteria to survive after cell invasion and grow in an adapted environment [70]. In the case of the *Erwinia *species which have been investigated, *cnf1*- and *cnf2*-genes could be identified in all species except *E. billingiae*, a possible role of Cnf may be to weaken defence response therefore helping the bacteria to spread. Cnf is thought to influence transcription factors via effects on signal transduction [71].

**Figure 5:**
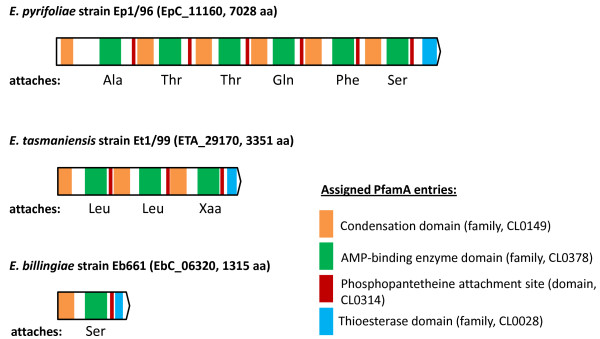
**Analysis of NRPSs for the strains Ep1/96 (position 1,277,774- 1,298,860), Et1/99 (pos. 3,243,033- 3,253,088) and Eb661 (pos. complement 749,230- 753,177)**. Locus tag and size are given in brackets. NRPSs were identified by BLASTP and domains were assigned according to Pfam. Predicted amino acids to be synthesized are noted, if a prediction was available. Abbreviations of amino acids: Ala, Alanine; Gln, Glutamine; Leu, Leucine, Phe, Phenylalanine; Ser, Serine; Thr, Threonine.

A striking feature of *E. pyrifoliae *strain Ep1/96 is the presence of an assumed non-ribosomal peptide synthetase (NRPS; suggested name EppT; EpC_11160) with a size of 7028 aa. It shows similarities to several non-ribosomal peptide synthetases (NRPSs). It shows high similarity with a protein of unknown function from *Photorhabdus luminescens *(syn. *Xenorhabdus luminescens*), an enterobacterial pathogen of insects and an NRPS of the distantly related soft rot pathogen *P. atrosepticum *SCRI1043 encoded in an island typical for horizontal gene transfer [39,72]. Similar NRPS proteins are also encoded in the genome of *E. tasmaniensis *strain Et1/99 and *E. billingiae *strain Eb661, but differ in size and domain content (Figure 5). According to a prediction program the NRPS-encoding ORF of *E. pyrifoliae *strain Ep1/96 contains six modules for the synthesis of a hexameric peptide with Ala-Thr-Thr-Gln-Phe-Ser [73]. The NRPS proteins in the genome of *E. billingiae *and *E. tasmaniensis *are considerably smaller with an encoding capacity for possible attachment of modified Leu-Leu-Xaa and Ser, respectively. No significant relation to the modules to the analogous *E. pyrifoliae *region can be deduced. The short NRPS-regions may only encode small modified peptides.

The presence of the *eppT *gene in strain Ep1/96 may be a result of an integration event as it is indicated by the phage integrase located upstream. Similar proteins could be identified in other plant pathogens such as *P. syringae *pv. *syringae*, which has been involved in synthesis of the phytotoxin syringomycin [74]. This list can be extended to several plant pathogens such as *Ralstonia solanacearum *and *Xanthomonas axonopodis *pv. *citri*, but also to the non-pathogenic species *Pseudomonas fluorescens*[39]. The NRPSs vary in size (e.g. 3351 aa for Et1/99 compared to 7028 aa for Ep1/96) [75]. The NRPS of *E. pyrifoliae *and the one of *P. atrosepticum *show a similar length (7028 aa/7523 aa) and nearly 6000 aa could be aligned with an identity of at least 41%.

## Conclusions

The comparative analysis of *E. billingiae *strain Eb661, *E. tasmaniensis *strain Et1/99 and *E. pyrifoliae *strain Ep1/96 highlights the different genome organization within this genus driven by recombination events. The genomes of these epiphytic and pathogenic bacteria show high accordance of single genes and gene clusters, which are potential virulence factors in pathogenic species. The difference in lifestyle may depend on protein secretion and invasion into plants. *E. billingiae *lacks any T3SS in contrast to *E. tasmaniensis *and *E. pyrifoliae*. Differences between both pathogenic *E. amylovora *and *E. pyrifoliae *and the non-pathogenic *E. tasmaniensis *include a lack of the HAE-region in the *hrp/hrc*-T3SS, of the SopA and PagC proteins and the presence of a VirK protein and putatively of Type I fimbriae. *E. billingiae *shows much larger variations, e.g. apparently more possibilities for biofilm formation and adhesion, no synthesis or utilization of levan, the absence of a non-ribosomal peptide synthetase similar to EppT and a T3SS with the HAE-region, the SopA protein and possesses a VirK protein. Therefore, those components probably represent factors to describe pathogenic and non-pathogenic *Erwinia *species. Factors such as synthesis of EPS and levan, utilization of sugar and sugar alcohols as well as expression of proteases and siderophores may be related to nutrient acquisition and to modulate plant defence.

The role of the NRPS in the pathogenic strain Ep1/96 remains unclear. A phytotoxin could be an advantage in weakening the plant during the colonization and can explain differences in the habitat of *Erwinia *species.

Virulence for *E. pyrifoliae *may depend on the factors summarized in this section. Accumulation in the genome of *E. pyrifoliae *could be interpreted as the result of an evolutionary adaptation process resulting in the specialization as a plant pathogen. The genetic distance between the pathogenic *Erwinia *species and *E. tasmaniensis *is small, and larger to *E. billingiae*, which has a tendency to invade necrotic tissue of plants. Several differences in genes and their expression apparently restrict *E. tasmaniensis *to exist as an epiphyte on plant surfaces. This enables the species to survive especially on flowers and supports its potential to compete with pathogens such as *E. amylovora *and possibly *E. pyrifoliae*.

## Methods

### Genome determination

*E. pyrifoliae *strain Ep1/96 (DSM 12162) and *E. billingiae *strain Eb661 (DSM 17872) were cultured as described previously [11,18]. DNA was isolated with the Genomic DNA kit (Qiagen, Hildesheim, Germany) according to the manufacturer's instructions. The genomic sequences were determined by whole genome shotgun sequencing using Sanger based sequencing technology and pyrosequencing.

The genomes of *E. pyrifoliae *strain Ep1/96 and *E. billingiae *strain Eb661 were covered by short insert shotgun libraries with 1.5 and 2.5 kb inserts and fosmid libraries with 37 kb inserts (CopyControl™ Fosmid Library Production Kit, Epicentre, Madison, U.S.A.) [76]. End-sequencing was performed on recombinant plasmids using BigDye 3.1 chemistry and 3730XL capillary sequencers (ABI, Darmstadt, Germany) resulting in a 9-fold sequencing coverage for strain Ep1/96 and a 11-fold sequencing coverage for strain Eb661. The high number of fosmid reads (24,252 and 7,096) resulted in one uncovered chromosomal region for strain Ep1/96 and a complete physical coverage by fosmid clones of the chromosome of strain Eb661. To reduce finishing experiments pyrosequencing was performed using the GS20 sequencer for strain Ep1/96 and the GS FLX platform for strain Eb661 (both 454 life science/Roche) resulting in an additional 25-fold (1,043,447 reads) and 8-fold (194,142 reads) sequencing coverage, respectively.

Data assembly was performed for strain Ep1/96 within two steps. GS20 data were initially assembled by Newbler (454 life science/Roche) and the resulting contigs were fragmented using PERL scripts resulting in overlapping sequence fragments, which were assigned as forward and reverse reads in fasta format and the corresponding fasta quality files. The thus obtained faked reads were assembled together with the Sanger derived processed reads using PhredPhrap http://www.phrap.org. GS FLX data and Sanger derived reads for strain Eb661 were assembled using the Celera assembler [77]. Assembled data for both projects were imported into Consed, edited and verified. Finishing experiments were performed by primer-walking on bridging clones and PCR products to improve sequence quality and gap closure [78].

### Genome annotation and comparison

Protein coding sequences were predicted by Glimmer3 and manually curated in Artemis [79,80]. Artemis was also used to calculate cumulative GC-skews [(G-C)/(G+C)] of the chromosomes. The genome sequences were annotated with HTGA [81]. Structural rRNAs and tRNAs were determined using RNAmmer and tRNAscan-SE [82,83]. Integrated bacteriophages were predicted by PhageFinder [84]. Furthermore, sequence analysis was improved using the RAST analysis platform [85]. NRPSs content was subsequently analysed by Pfam http://pfam.sanger.ac.uk/ and specificity prediction (NRPSredictor, http://www-ab.informatik.uni-tuebingen.de/toolbox/) [73].

Genome comparisons were performed for *E. billingiae *strain Eb661 (this study), *E. tasmaniensis *strain Et1/99, *E. pyrifoliae *strain Ep1/96 (this study) and partial sequences of *E. amylovora *strain Ea273 [15]. The complete genome sequence of *E. amylovora *strain Ea273 (ATCC 49946) was published recently: FN666575, FN666576, and FN666577. Conserved synteny was examined using ACT with the big_blast.pl script (5000 bases sequence size; BLASTN algorithm) [86].

Core and pan genome for the deduced proteins were analysed by BLASTCLUST http://www.ncbi.nlm.nih.gov/ and parsed through a script to obtain intersections. Numbers were calculated using an identity of 60%, an alignment length of 70% and an e-value of 1e-6 at least as cutoffs for these analyses. The utilized scripts were implemented in a new public web-service at http://www.reziclust.molgen.mpg.de, which allows recalculation of results as well as performing new analyses. Results were parsed in addition through scripts for visualization of the present or absent potential orthologs within each genome.

Potential paralogs were estimated using BLAST [87]. Deduced protein sets of the chromosomes were compared against themselves and filtered with MSPcrunch using a minimal identity of 90% for this purpose [88].

### Data and strain access

The annotated sequences of *E. pyrifoliae *strain Ep1/96 have been deposited in Genbank/EMBL/DDBJ under accession numbers FP236842 (chromosome), FP928999 (pEp2.6), FP236827 (pEP03), FP236828 (pEP05), FP236829 (pEP36), and of *E. billingiae *strain Eb661 under accession numbers FP236843 (chromosome), FP236826 (pEB102) and FP236830 (pEB170).

The strains used for genomic sequencing are deposited in various international strain collections: Ep1/96 as DSM 12162, CFBP 4171; Eb661 (type strain) as DSM 17872, CFBP 6830, NCPPB 661, LMG 2613 and the previously sequenced *E. tasmaniensis *strain Et1/99 (type strain) as DSM 17950, CFBP 7177, NCPPB 4357, LMG 25318.

## Authors' contributions

MK, AMM, HK, KH and RR contributed to library construction, template preparation, sequence determination and assembly. AMM, IG, FK, YM, KG and MK participated in annotation and performed sequence analysis. MK, AMM, KG and RR drafted the manuscript.

All authors read and approved the final manuscript.

## Supplementary Material

Additional file 1 **Cumulative GC skew [(G-C)/(G+C)] of three investigated chromosomes**. Click here for file

Additional file 2 **Chromosome maps of the three investigated species highlighting the conserved protein-coding gene content and the individual set**. PDFs are of high resolution and allow enlarging regions of interest. Click here for file

Additional file 3 **Genes with potential impact for virulence in pathogenic erwinias encoded in the genomes of *E. pyrifoliae*, *E. billingiae *and *E. tasmaniensis***. Click here for file

Additional file 4 **A scheme for involvement of proteins from *E. pyrifoliae*, *E. tasmaniensis *and *E. billingiae *or in assembly of the T6SS**. Click here for file
