# Supplementary material for: Genome comparison of the epiphytic bacteria Erwinia billingiae and E. tasmaniensis with the pear pathogen E. pyrifoliae
Source: BMC Genomics. 2010 Jun 22;11:393. doi: 10.1186/1471-2164-11-393 (PMC2897811; doi:10.1186/1471-2164-11-393)
Supplement: Additional file 1 — Cumulative GC skew [(G-C)/(G+C)] of three investigated chromosomes. [file 1471-2164-11-393-S1.PDF]

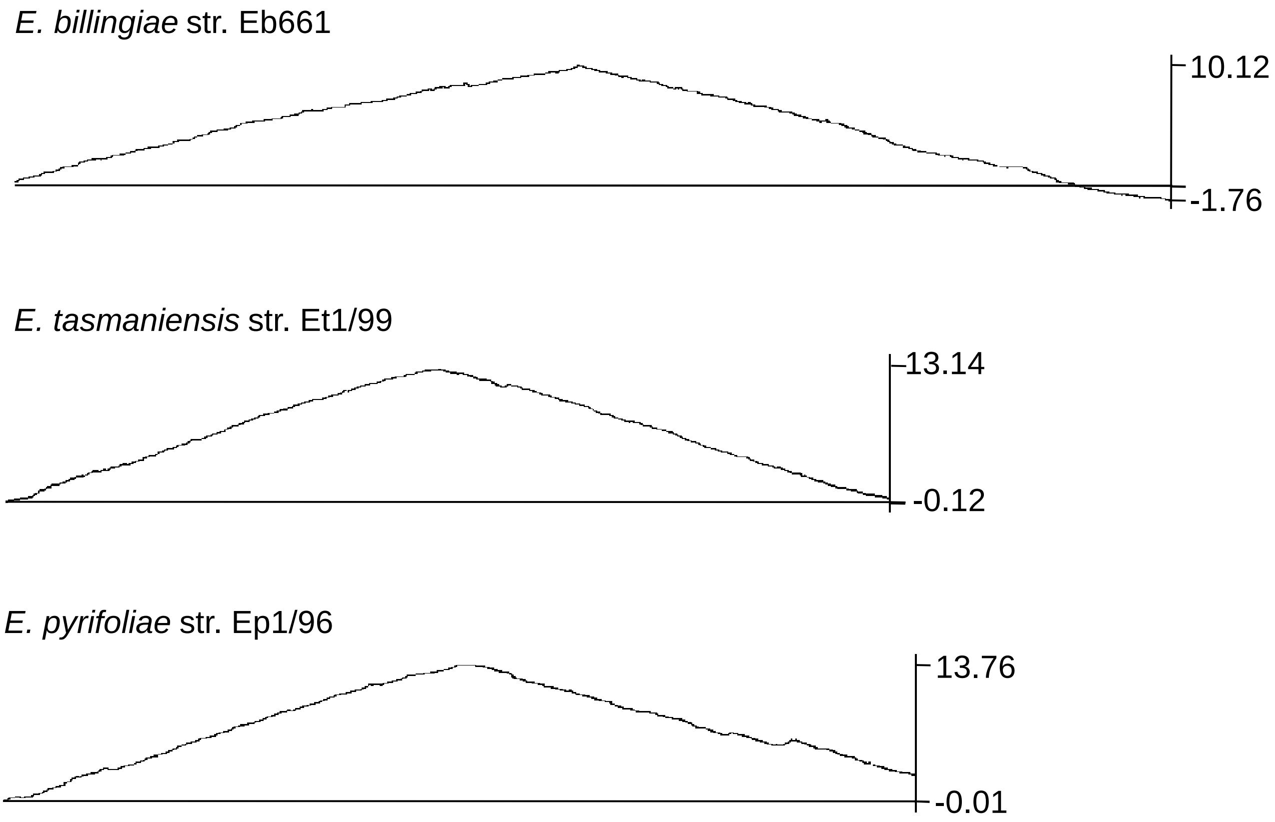

Additional file 1.
Cumulative GC skew [(G-C)/(G+C)] of three investigated chromosomes. A window size of 5000 bases was used for calculation. Maximal and minimal values are indicated.
