# Supplementary material for: Genome comparison of the epiphytic bacteria Erwinia billingiae and E. tasmaniensis with the pear pathogen E. pyrifoliae
Source: BMC Genomics. 2010 Jun 22;11:393. doi: 10.1186/1471-2164-11-393 (PMC2897811; doi:10.1186/1471-2164-11-393)
Supplement: Additional file 2 — Chromosome maps of the three investigated species highlighting the conserved protein-coding gene content and the individual set. PDFs are of high resolution and allow enlarging regions of interest. [file 1471-2164-11-393-S2.PDF]

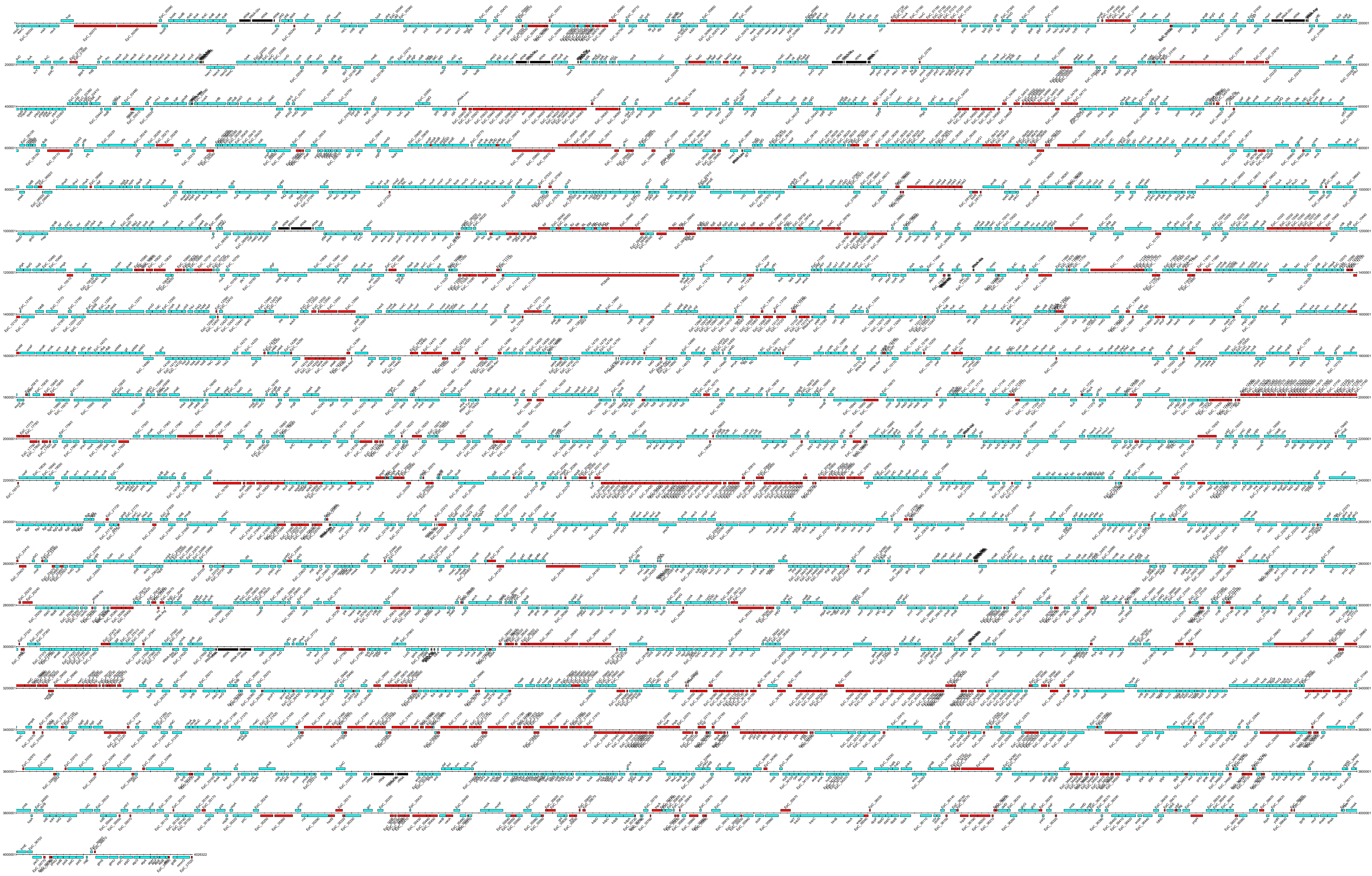

Figure S2A, *E. pyrifoliae* strain Ep1/96.

Genes (protein-coding) present on the chromosome of *E. pyrifoliae,* but absent on the chromosomes of *E. tasmaniensis* strain Et1/99 and *E. billingiae* strain Eb661, are highlighted in red.

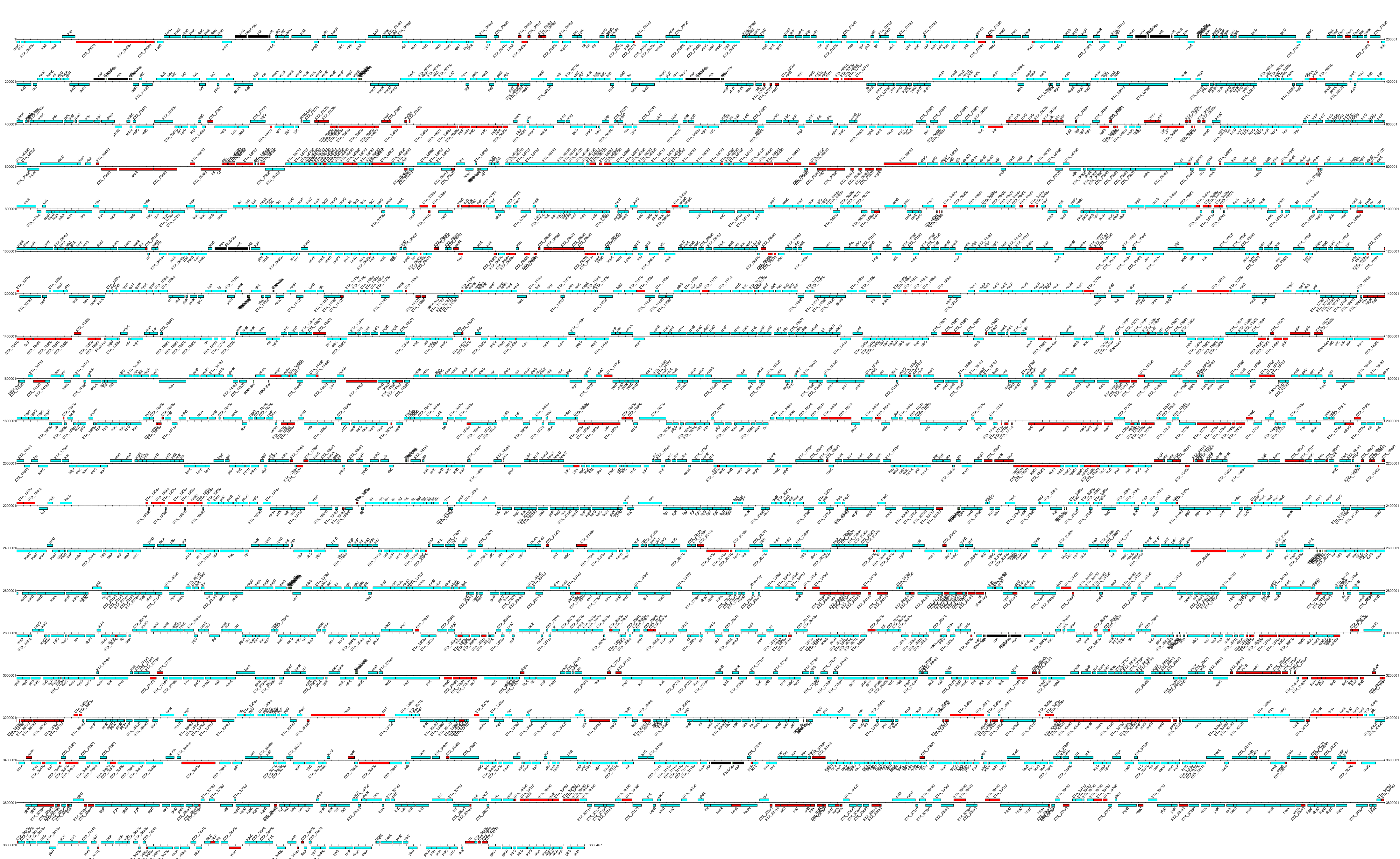

Figure S2B, *E. tasmaniensis* strain Et1/99.

Genes (protein-coding) present on the chromosome of *E. tasmaniensis,* but absent on the chromosomes of *E. pyrifoliae* strain Ep1/96 and *E. billingiae* strain Eb661, are highlighted in red.

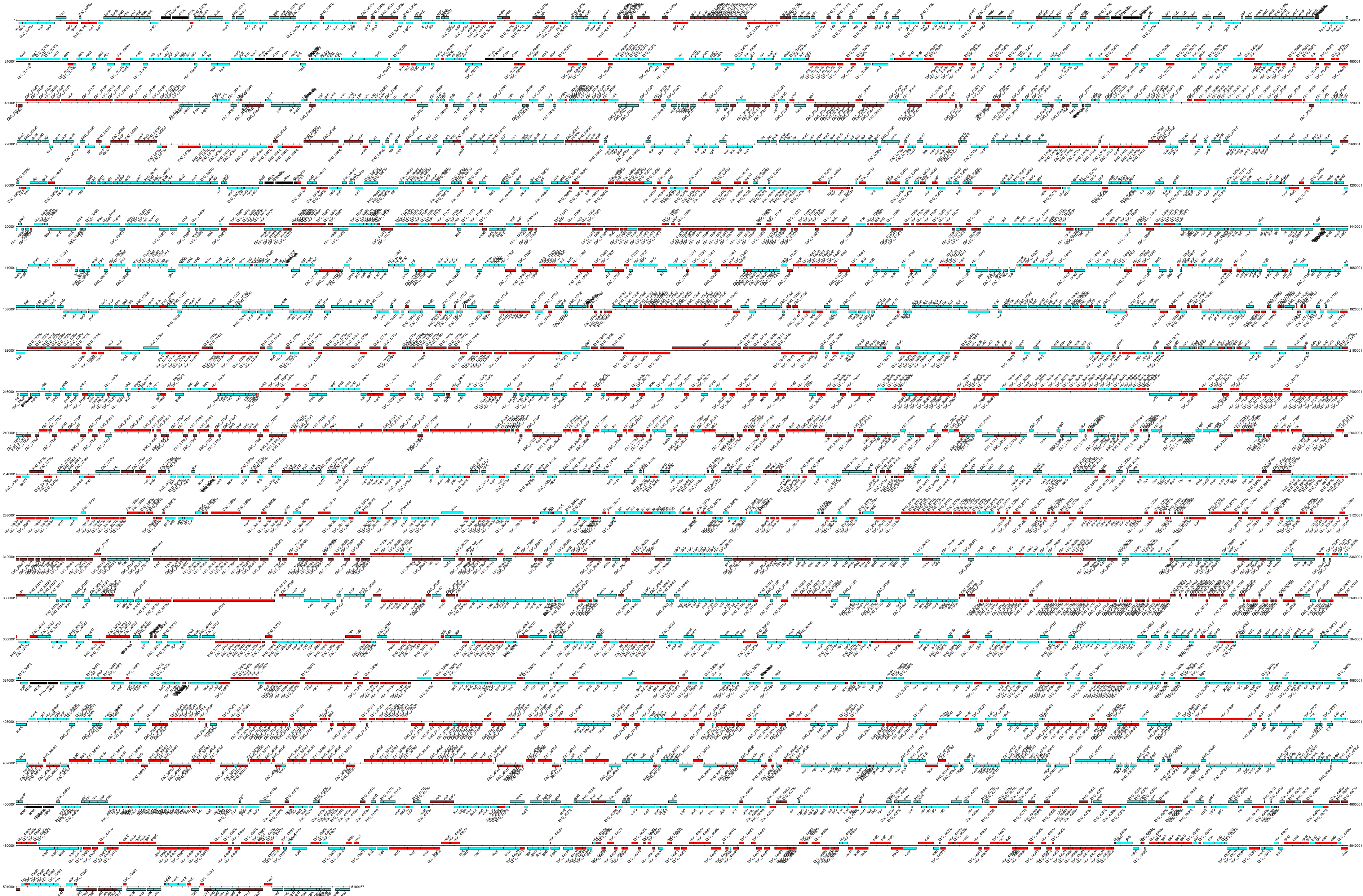

Figure S2C, *E. billingiae* strain Eb661.

Genes (protein-coding) present on the chromosome of *E. billingiae,* but absent on the chromosomes of *E. tasmaniensis* strain Et1/99 and *E. pyrifoliae* strain Ep1/96, are highlighted in red.

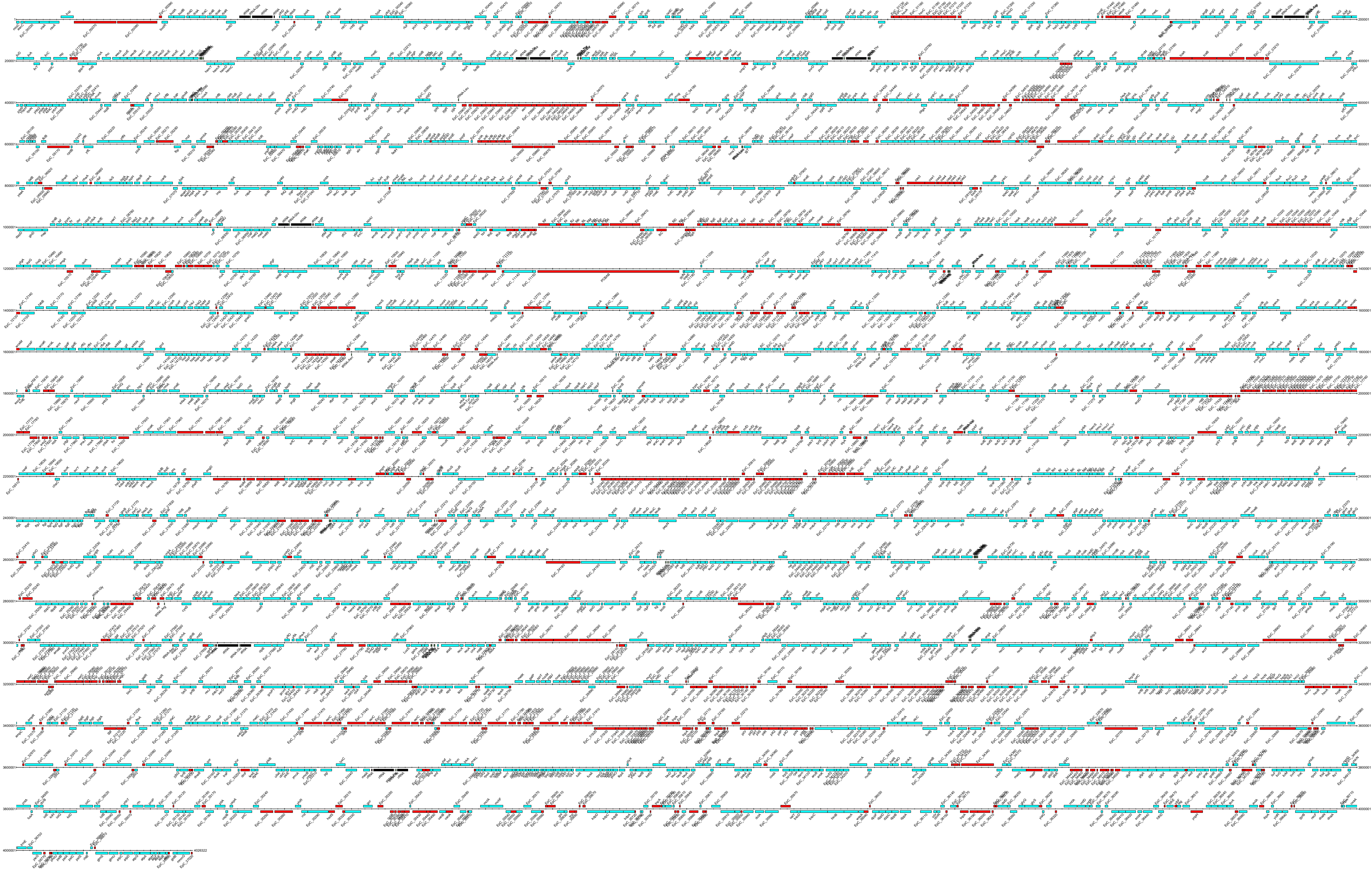

Figure S2D, *E. pyrifoliae* strain Ep1/96.

Genes (protein-coding) present on the chromosome of *E. pyrifoliae* but absent on the chromosome of *E. tasmaniensis* strain Et1/99 are highlighted in red.

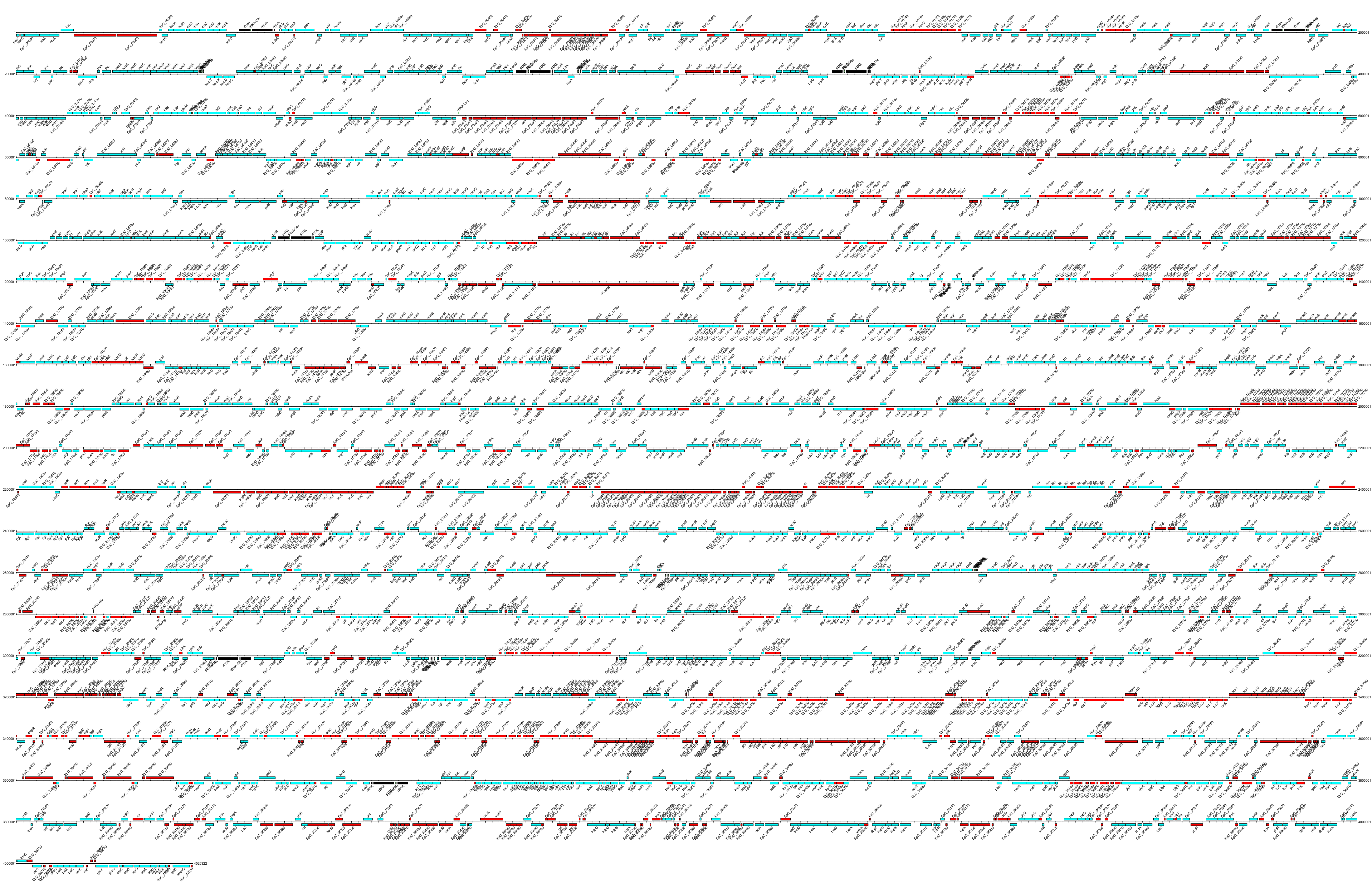

Figure S2E, *E. pyrifoliae* strain Ep1/96.

Genes (protein-coding) present on the chromosome of *E. pyrifoliae* but absent on the chromosome of *E. billingiae* strain Eb661 are highlighted in red.
