# Supplementary material for: Genome comparison of the epiphytic bacteria Erwinia billingiae and E. tasmaniensis with the pear pathogen E. pyrifoliae
Source: BMC Genomics. 2010 Jun 22;11:393. doi: 10.1186/1471-2164-11-393 (PMC2897811; doi:10.1186/1471-2164-11-393)
Supplement: Additional file 3 — Genes with potential impact for virulence in pathogenic erwinias encoded in the genomes of E. pyrifoliae, E. billingiae and E. tasmaniensis. [file 1471-2164-11-393-S3.PDF]

Additional file 3.

Candidate genes with potential impact for virulence in pathogenic erwinias encoded in the genomes of *E. pyrifoliae*, *E. billingiae* and *E. tasmaniensis*. If no gene name could be assigned, the corresponding locus tags from the *E. pyrifoliae* are provided. Names of the genes encoding proteins with the potential to act as effectors made bold.

**Secretion systems and effectors**					
**Functional system**	**Product**	**Gene name**	**Ep1/96**	**Et1/99**	**Eb661**
hrp/hsv/dsp cluster (T3SS)	Type III effector	***hrpK***	+	-	-
	Hrp-associated systemic virulence proteins	*hsvA*	+	-	-
		hsvB	+	-	-
		hsvC	+	-	-
	Hrp/hrc secretion/translocation pathway proteins	*hrcU*	+	+	-
		hrcT	+	+	-
		hrcS	+	+	-
		hrcR	+	+	-
		hrcQ	+	+	-
		hrpP	+	+	-
		hrpO	+	+	-
		hrcN	+	+	-
		hrpQ	+	+	-
		hrpI/hrcV	+	+	-
		hrpJ	+	+	-
		hrpL	+	+	-
		hrpX	+	+	-
		hrpY	+	+	-
		hrpS	+	+	-
		hrpA	+	+	-
		hrpB	+	+	-
		hrcJ	+	+	-
		hrpD	+	+	-
		hrpE	+	+	-
		hrpF	+	+	-
		hrpG	+	+	-
		hrcC	+	+	-
		hrpT	+	+	-
		hrpV	+	+	-
	Hrp elicitor/effector region	***hrpN***	+	+	-
		orfA	+	+	-
		orfB	+	+	-
		orfC	+	+	-
		hrpW	+	+	-
		dspA/E	+	+	-
	Chaperone for DspA/E	*dspB/F*	+	+	-
Salmonella SPI-1-like Type III secretion system and related exported proteins	Cell adherance/invasion protein	*InvH*	-	-	-
	Secretion chaperone	*invF*	+	+	-
	Secretion protein	*invG*	+	+	-
	Cell invasion protein	*invE*	+	+	-
	Invasion protein	*invA*	+	+	-
	Surface presentation of antigens	*invB*	+	+	-
	ATPase	*invC/spaL*	+	+	-

	Surface presentation of antigens	*invI/spaM*	-	-	-
		invJ/spaN	-	-	-
		spaO	+	+	-
		spaP	+	+	-
		spaQ	+	+	-
		spaR	+	+	-
		spaS	+	+	-
	Secretion protein	*spaT*	-	-	-
	Chaperone protein (*Salmonella* invasin chaperone)	*sicA*	+	+	-
	Cell invasion protein	***sipB***	+	+	-
		sipC	-	-	-
		sipD	+	+	-
		sipA	-	-	-
	Protein tyrosine phosphatase	*sptP*	-	-	-
	Cell invasion proteins	*prgH*	+	+	-
		prgI	+	+	-
		prgJ	(+)	(+)	-
		prgK	+	+	-
	Oxygen-regulated invasion protein	*orgA*	+	+	-
	Invasion plasmid antigen	***ipaB***	-	-	-
	Cell invasion protein	***ipaC***	-	-	-
	Invasion plasmid antigen	*ipaD*	-	-	-
	Invasin	*ipaA*	-	-	-
Type V secretion system	Autotransporter/effector proteins	Porin-/Pertactin-like	-	-	+
Type VI secretion system	Lipoprotein VCA0113	EpC_06160	+	+	+
	conserved uncharacterized protein	EpC_06170	+	+	+
	OmpA/MotB-like protein	*vasF1*	+	+	+
	IcmF-like protein	EpC_06190	+	+	+
	T6SS associated protein	EpC_06200	+	+	+
	T6SS associated ImpA protein	EpC_06210	+	+	+
	conserved uncharacterized protein	EpC_06220	+	+	+
	conserved uncharacterized protein	EpC_06230	+	+	+
	conserved uncharacterized protein	EpC_06240	+	+	-
	Virulence factor for secretion apparatus	*hcp*	+	+	+
	putative exported protein	EpC_06290	+	-	+
	FHA domain-containing protein	EpC_06300	+	+	+
	Protein phosphatase 2C-like protein	EpC_06310	+	+	+
	conserved uncharacterized protein	EpC_06320	+	+	+
	conserved uncharacterized protein	EpC_06330	+	+	+
	conserved uncharacterized protein	EpC_06340	+	+	+
	T6SS family protein, VCA0110	*vasA*	+	+	+
	conserved uncharacterized protein	EpC_06360	+	+	+
	conserved uncharacterized protein	EpC_06370	+	+	-
	conserved uncharacterized protein	EpC_06380	+	+	-
	T6SS family ATPase. ClpV1	EpC_06390	+	+	+
	Serine/threonine protein kinase	EpC_06400	+	+	+
	Rhs element Vgr protein	*vgrG1*	+	+	+
	Rhs element Vgr protein	*vgrG2*	+	+	+
	Putative invasin YchP	*ychP*	+	+	+
	OmpA family protein	*vasF2*	+	+	+
	IcmF protein	EpC_19530	+	+	+
	T6SS associated protein	EpC_19540	+	+	+
	FHA domain-containing protein	EpC_19550	+	-	+
Effector and virulence proteins	SsrAB regulated protein	***srfA***	+	+	+
	Virulence protein	***srfB***	+	+	+
	Virulence effector protein	***srfC***	+	+	+

	Secreted effector protein	***sopA***	+	-	-
	maintenance of virulence plasmid, toxin	*mvpT*	+	+	-
	Virulence protein	*msgA*	+	+	+
	Two-component response regulator of virulence determinants	*phoP*	+	+	+
	Virulence sensor histidine kinase	*phoQ*	+	+	+
	mouse virulence protein	*mviN*	+	+	+
	Virulence-related outer membrane protein X	*ompX*	+	+	+
	Virulence outer membrane protein	*pagC*	+	-	+
	Putative virulence protein	*virK*	-	+	+
	Putative tyrosine-protein phosphatase YopH (Virulence protein)	***yopH***	+	+	-
**Metabolism**					
**Functional system**	**Product**	**Gene name**	**Ep1/96**	**Et1/99**	**Eb661**
Capsular polysaccharide biosynthesis	UDP-galactose-lipid carrier transferase	*ams/cpsG*	+	+	+
	Periplasmic protein involved in polysaccharide export	*ams/cpsH*	+	+	+
	Low molecular weight protein-tyrosine-phosphatase	*ams/cpsI*	+	+	+
	Tyrosine-protein kinase	*ams/cpsA*	+	+	+
	Glycosyltransferase	*ams/cpsB*	+	+	+
	Exopolysaccharide biosynthesis protein	*ams/cpsC*	+	+	+
	Glycosyltransferase	*ams/cpsD*	+	+	+
		ams/cpsE	+	(+)	(+)
	Exopolysaccharide biosynthesis proteins	*ams/cpsF*	+	+	+
		ams/cpsJ	+	+	+
	Glycosyltransferase	*ams/cpsK*	+	+	+
	Exopolysaccharide biosynthesis protein	*ams/cpsL*	+	+	+
	Possible subunit of GalU	*galF*	+	+	+
	UDP-glucose 4-epimerase	*galE*	+	+	+
	Putative capsular polysaccharide lipoproteins	*ymcA*	+	+	+
		ymcB	+	+	+
		ymcC	+	+	+
		rcsA	+	+	+
	Activator of capsular EPS synthesis	*rcsB*	+	+	+
	Regulator of capsular EPS synthesis	*rcsC*	+	+	+
		rcsD	+	+	+
Levan metabolism	Levansucrase	*lsc*	-	+	+
	Levanase	EpC_17920	+	-	-
	Regulators of levansucrase expression	*rlsA*	+	+	-
		rlsB	+	+	-
		rlsC	+	+	-
Sorbitol-Operon	Glucitol/sorbitol-specific enzyme IIC	*srlA*	+	-	+
	Enzyme IIB of PTS	*srlE*	+	-	+
	Enzyme IIA of PTS	*srlB*	+	-	+
	Sorbitol-6-phosphate 2-dehydrogenase	*srlD*	+	-	+
	Activator of *srl* operon	*srlM*	+	-	+
	Repressor of *srl* operon	*srlR*	+	-	+
Sucrose metabolism	Fructokinase	*srcK*	+	+	-
	Porin	*srcY*	+	+	-
	Enzyme II	*srcA*	+	+/+	-
	Hydrolase	*srcB*	+	+/+	-
	Repressor	*srcR*	+	+	-
NRPS	Non-ribosomal peptide synthetase	*eppT*	+	(+)	-

**Adhesion and extracellular factors**					
**Functional system**	**Product**	**Gene name**	**Ep1/96**	**Et1/99**	**Eb661**
Necrosis factors	Probable cytotoxic necrotizing factor 1	*cnf1*	+	+	-
	factor 2	*cnf2*	+	+	-
Proteases	Protease III	*ptrA*	+	+	+
	Protease II	*ptrB*	+	+	+
	Protease A of *E. amylovora*	*prtA,D,E,F*	-	-	-
Siderophores	Ferrioxamine receptor	*foxR*	+	+	+
	Siderophore biosynthetis enzyme, L-lysine 6-monooxygenase (NADPH)	*dfoA*	+	+	-
	Siderophore biosynthesis protein, probable alcaligin	*alcA*	+	+	-
