# Supplementary material for: Genome comparison of the epiphytic bacteria Erwinia billingiae and E. tasmaniensis with the pear pathogen E. pyrifoliae
Source: BMC Genomics. 2010 Jun 22;11:393. doi: 10.1186/1471-2164-11-393 (PMC2897811; doi:10.1186/1471-2164-11-393)

Additional file 4. A scheme for involvement of proteins from *E. pyrifoliae*, *E. tasmaniensis* and *E. billingiae* in assembly of the T6SS.

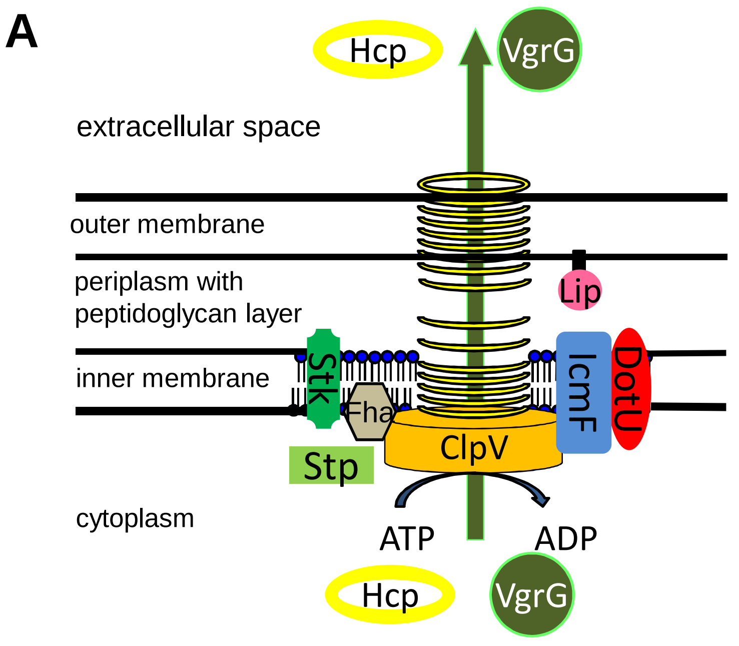

A. The model with basic components of the T6SS was adapted from Filloux et al. (2008). The ClpV ATPase (orange) may be involved in the transport of Hcp (yellow) and VgrG (dark green) across the cell envelope. Hcp proteins are supposed to be secreted independently. VgrG may be released into the eukaryotic cell or to the bacterial cell surface. Lip (pink) acts as putative outer-membrane lipoprotein. IcmF (blue) and DotU (red) are inner-membrane proteins, probable interacting with ClpV. The level of phosphorylation of the Fha protein (brown) regulates T6SS activity. Stk (green) is the Ser/Thr kinase and Stp (light green) represents the corresponding Ser/Thr phosphatase. **B.** T6SS region of the analyzed strains show a conserved gene order and content for the genes assigned to the T6SS. Genes only assigned by the usage of TIGRFAM are highlighted (grey). Genes without functional assignment to T6SS are not colored. All strains encode for a second island associated to T6SS with weak identities to the other and differing in gene content at least. The assignment of a second putative lipoprotein in strain Ep1/96 also remains unclear. The absence of the putative secreted *vgrG* gene within the first T6SS region of strain Eb661 could be the result of a translocation because *vgrG*-like genes as well *clpV*-like genes occur scattered over the chromosome.

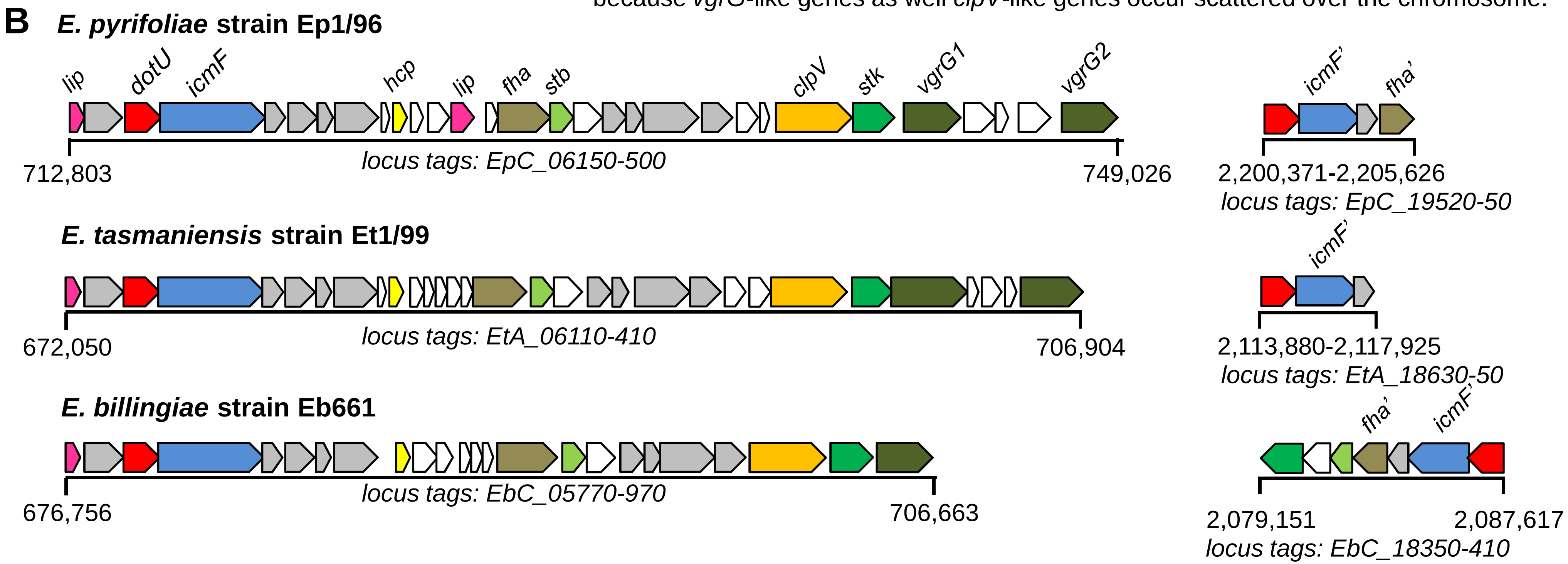

Supplement: Additional file 4 — A scheme for involvement of proteins from E. pyrifoliae, E. tasmaniensis and E. billingiae or in assembly of the T6SS. [file 1471-2164-11-393-S4.PDF]
